# Diagnosis of brain death in wistar rats at different levels of death induction

**DOI:** 10.12688/f1000research.157233.1

**Published:** 2024-12-06

**Authors:** Nayara Maria Gil Mazzante, Fernanda Zuliani, Rogerio Antonio de Oliveira, Júlia Soares Bodaneze, Giovanna Farina Panebianco, Natália Freitas de Souza, Fernando Carmona Dinau, Paola Alejandra Montenegro Cuellar, Nadia Yumi Yamamo dos Santos, Ana Beatriz de Souza da Silva, Fernanda de Freitas Alves Vieira, Natália Camargo Faraldo, Gabriela Abreu Botelho, Fernanda Barthelson Carvalho de Moura, Noeme Sousa Rocha

**Affiliations:** 1 Department of Veterinary Clinics, School of Veterinary Medicine and Animal Science, São Paulo State University - Botucatu Campus, Botucatu, State of São Paulo, Brazil; 2 Department of Biostatistics, Institute of Biosciences, São Paulo State University (UNESP), Botucatu, State of São Paulo, Brazil; 3 Department of Veterinary Surgery and Reproduction, School of Veterinary Medicine and Animal Science, São Paulo State University, Botucatu, State of São Paulo, Brazil

**Keywords:** brain death; hematologic analysis; Wistar rats

## Abstract

**Objective:**

This study aimed to evaluate hematologic, biochemical, and gasometric parameters in Wistar rats to better understand brain death parameters and reduce early misdiagnoses.

**Methods:**

Fifteen adult male Wistar rats (Rattus norvergicus; HanUnib: WH) were randomly distributed into three groups of five animals: the control group (G0) with evaluation performed before brain death, and two groups (G1 and G2) with brain death induced at different times: immediately after induction (G1) and one hour after induction (G2). Venous and arterial blood samples were taken to perform complete blood count, biochemical, and blood gas assays. Samples were taken at specific times based on the group each rat belonged to.

**Results:**

Statistically significant mean values were observed (P<0,05) for segmented cells (G1>G2 and G0>G2), monocytes (G2>G1 and G0>G1), creatinine (G2>G0), aspartate aminotransferase (G1>G0), potassium (G2>G0), and bicarbonate (G0>G1).

**Discussion:**

Furthermore, brain death showed a unique response in each organism, complicating its precise determination even more.

## Introduction

Brain death (BD), the complete and irreversible loss of brain function, is a complex process characterized by an inflammatory state leading to cellular and molecular disturbances that alter the physiology and biochemistry of the organic system. ^1^ However, criteria for determining BD vary among countries, each having national laws or guidelines, resulting in a lack of standardization. In 50% of countries, clinical examination is sufficient to determine BD, whereas the other 50% require complementary tests. ^2^

Currently, the American Association of Neurology (AAN) defines BD with three cardinal signs: interruption of brain functions, including the brainstem, coma or unresponsiveness, and apnea. ^3^ In Brazil, social changes and advancements in medicine led the Federal Council of Medicine (CFM) to update the criteria for diagnosing BD in 2017, with Resolution No. 2,173 of November 23, replacing No. 1,408/97. The changes included specific physiological prerequisites for patients, requirements for doctors to provide care before diagnosing BD, the necessity of complementary tests, and specific training for doctors making this diagnosis. ^4, 5^

Determining the moment of BD is complicated by significant complex pathophysiological changes involving excitation of the sympathetic nerves, hormonal imbalance, hemodynamic instability, and metabolic disorders with the release of cytokines, ^6^ which peak at alternating times due to the type of reaction and injury severity. ^7, 8^ During BD progression, a systemic inflammatory response can worsen, leading to disseminated intravascular coagulation mediated by inflammatory mediators from the ischemic brain, ischemic reperfusion injury, metabolic changes during the catecholamine storm, and an inadequately restored cardiovascular state. This also produces sudden changes in blood pressure, hypoxemia, hypothermia, coagulopathy, and electrolyte and hormonal disorders. ^9, 10^

More studies are needed to accurately determine the moment of BD, especially due to the scarcity of data. The literature on BD in animals is even more limited. Given the social importance of animals and the growing demand for scientific data to determine and establish death in animals, this has become an important issue for veterinarians. This importance is underscored by the increase in the animal market (farming, pet market, etc.) and the evolution of veterinary medicine (freelancers, clinics, hospitals, and research centers). Therefore, this study aimed to evaluate the hematological, biochemical, and gasometric parameters in rats, as these can be determinants of BD and help elucidate its concept, reducing errors in early diagnosis.

## Methods

Fifteen Wistar adult male rats (Rattus norvegicus) of the HanUnib: WH line were acquired from the Multidisciplinary Center for Biological Research at the State University of Campinas (CEMIB - UNICAMP). They were housed in the Central Experimental Vivarium of the Experimental Research Unit (UNIPEX) at the Medical School (FMB) of São Paulo State University (UNESP). The rats were kept in polypropylene cages with metal grid covers in a room maintained at 22 °C and 55% humidity under a light-dark cycle. The protocols used in this study are in accordance with the Ethical Principles in Animal Research adopted by the Brazilian College of Animal Experimentation (COBEA) and were approved by the Ethics Committee on the Use of Animals (CEUA) of the Faculty of Medicine (FMB) of UNESP, Botucatu Campus, protocol No. 0259/2018 on 16 ^th^ January of 2019.

Food and water were provided ad libitum, and environmental enrichment was performed using polyvinyl chloride (PVC) pipes and paper balls during the experimental period. Individual body weights were recorded weekly to monitor hygiene, adjust the number of animals per cage, and ensure their welfare. After an acclimatization period of 2 weeks, the animals were randomly divided into three groups, each with five animals: control group (G0) (n=5), evaluated before BD, and two groups with BD induction, immediately after induction (G1) (n=5) and 1 h after induction (G2) (n=5).

Before the induction of BD, the animals were anesthetized, initially in an induction box with isoflurane (9–5%) and then with an isoflurane mask (5–2.5%). Intubation was performed using a 14G or 16G catheter, and rats were maintained in the inhalation anesthesia circuit with isoflurane (3–1.5%). Trichotomy and asepsis were performed using chlorhexidine degermante and 70% alcohol in the area of access to the aorta and femoral vein. A 24G catheter was used to access the aorta and femoral vein, and a three-way tap circuit was set up to monitor blood pressure and collect blood for blood gas analysis, along with venous blood collection for blood count and biochemical tests. Animals with mean arterial pressure (MAP) >70 mmHg were used for hematological, biochemical, and hemogasometric parameters. Those without stable MAP were excluded from the experiment.

Blood samples were collected from each animal according to their respective group timings: G0 at time 0 (M0, before BD), G1 at time 1 (M1, shortly after BD induction), and G2 at time 2 (M2, 1 h after BD induction). Venous blood samples were drawn from the femoral vein into 0.5 ml ethylenediaminetetraacetic acid (EDTA) tubes and refrigerated (6–10 °C) for subsequent erythrogram examination (including red blood cells [RBC], hemoglobin [Hb], hematocrit [HCT], mean corpuscular volume [MCV], mean corpuscular hemoglobin concentration [MCHC], mean corpuscular hemoglobin [MCH], total plasma protein [TPT], red cell distribution width [RDW], and platelets) and leukogram (covering total leukocytes, neutrophils, segmented, lymphocytes, eosinophils, and monocytes).

Processing was performed using a Hemacounter 60-RT 7600 hematological analyzer (Hemogram, China). Hematocrit was determined by centrifuging microhematocrit samples at 12,000 rpm for 5 min. Platelet counts were performed manually by diluting 20 μL of blood in 2 mL of Brecher solution and counting in a Neubauer chamber. The differential leukocyte count was executed on panotype-stained blood smears (Laborclin) under an optical microscope with immersion (1,000× magnification).

The biochemical examination was conducted using the BS200E Analyzer (Mindray, Brazil). Venous blood samples from the femoral vein were collected in dry tubes, refrigerated (6–10 °C), and centrifuged at 2,500 rpm for 5 min. This process was followed by analyzing urea, creatinine (Cr), alanine aminotransferase (ALT), aspartate aminotransferase (AST), alkaline phosphatase (ALP), gamma-glutamyl transferase (GGT), serum total protein (TP), albumin, globulin, creatine kinase (CK), and CK-MB.

Blood gas analysis was performed using the ABL80 Flex Analyzer - BASIC Version (RADIOMETER). Arterial blood samples were collected from the aortic artery in 1 mL heparinized syringes (volume collected 0.7 mL), immediately packed in a styrofoam container with crushed ice, and analyzed for blood gases (pH, pCO _2_, and pO _2_), electrolytes, and metabolites (Na ^+^, K ^+^, Ca ^2+^, Cl ^-^, Lac), and derived values (HCO _3_ and sO _2_).

BD induction followed the protocol described by Esmaeilzadeh et al. ^10^ A Fogarty 14G catheter (Baxter Health Corp., CA, USA) was inserted via frontolateral trepanation into the skull using a surgical drill. Intracranial pressure (ICP) was increased by slowly inflating with 400–700 μL of saline. The catheter balloon inflation began with 100 μL of saline solution, and after 1 min, an additional 100 μL of solution was added. This procedure was repeated until the absence of corneal reflexes confirmed the BD, maximally dilated fixed pupils and 60 s apnea.

Following BD confirmation, all animals had their body temperature maintained using a thermal mattress, and blood pressure (BP) and heart rate (HR) were continuously monitored with a multiparameter monitor, adjusted according to the respective group. Animals in the BD-induced groups were supported with mechanical ventilation after anesthesia withdrawal. After BD, the animals received a heated infusion of sodium chloride 0.9% supplemented with potassium chloride at a rate of 3–5 mL/kg/h, along with dopamine infusion (5–15 mcg/kg/min), with bolus administration as needed, to conclude animals’ euthanasia.

## Results

**Figure 1.**
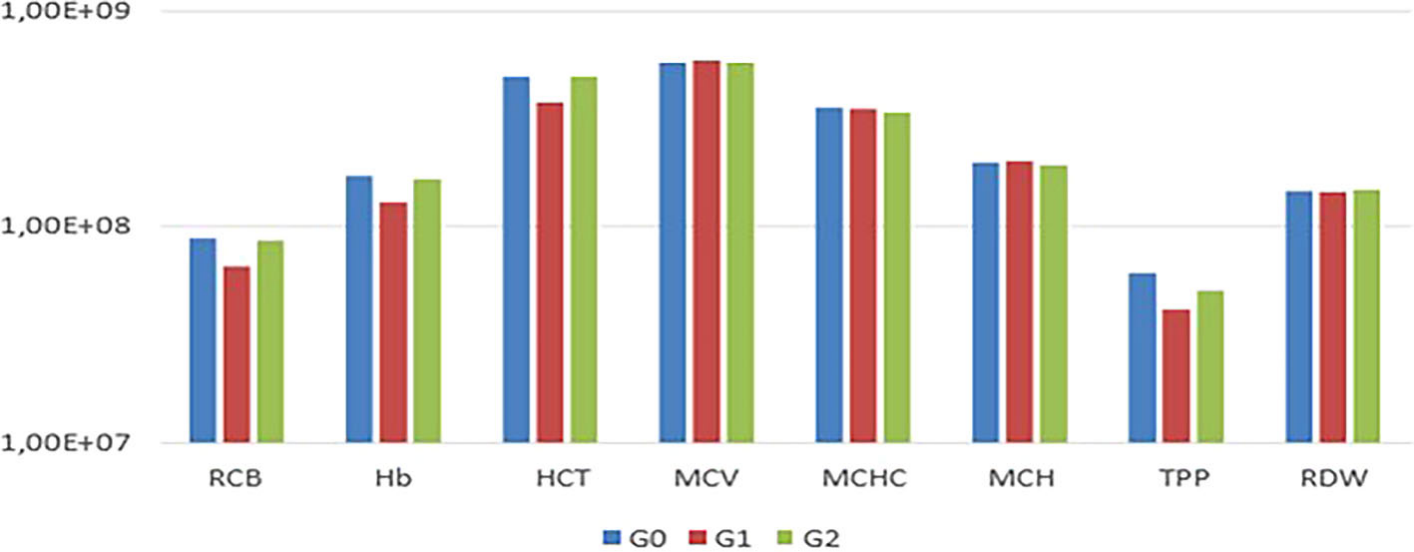
Comparison of mean erythrogram values among G0, G1, and G2, with no statistical differences observed (p<0.05).

**Figure 2.**
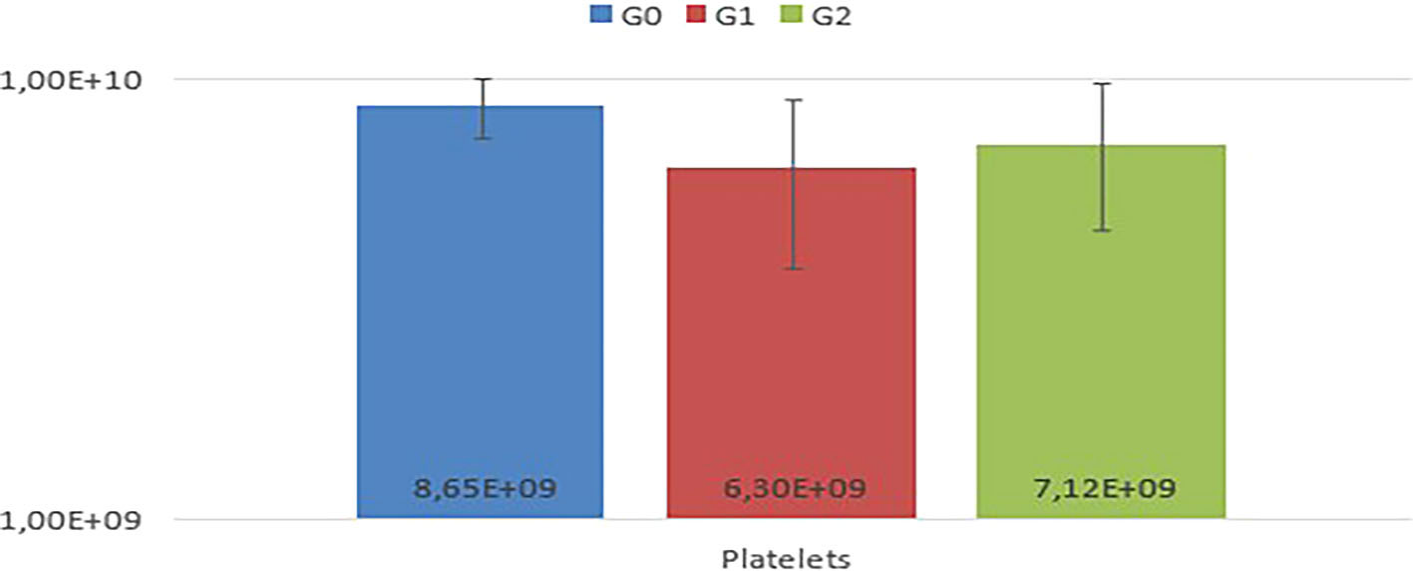
Comparison of mean platelet values among G0, G1, and G2, with no statistical differences observed (p<0.05).

In the comparison among G0, G1, and G2, no statistically significant differences (P<0.05) were observed in the mean values of RBC, Hb, HCT, MCV, MCHC, MCH, TPP, RDW, and platelets among the experimental animals ( Table 1, Figures 1 and 2).

**Table 1.** Comparisons between groups 0, 1 and 2 of the values for Red Blood Cells (RBC), Hemoglobin (Hb), Hematocrit (HCT), Mean Corpuscular Volume (MCV), Mean Corpuscular Hemoglobin Concentration (MCHC), Mean Corpuscular Hemoglobin (MCH), Total Plasma Protein (TPP), Red Cell Distribution Width (RDW) and Erythrogram Platelets obtained from the experimental animals. Differences in group least squares means, adjusted for multiple comparisons - Tukey-Kramer of the variables.

Variable	Group	_Group	Estimate	Standart Error	Value z	Pr˃│z│	Adj p
RBC	1	2	-0.2860	0.1385	-2.06	0.0390	0.0973
RBC	1	0	-0.2953	0.1469	-2.01	0.0445	0.1098
RBC	2	0	-0.00930	0.1469	-0.06	0.9495	0.9978
Hb	1	2	-0.2579	0.1354	-1.91	0.0568	0.1372
Hb	1	0	-0.2765	0.1436	-1.93	0.0542	0.1315
Hb	2	0	-0.01857	0.1436	-0.13	0.8971	0.9908
HCT	1	2	-0.2850	0.1295	-2.20	0.0278	0.0710
HCT	1	0	-0.2706	0.1373	-1.97	0.0488	0.1195
HCT	2	0	0.01433	0.1373	-0.10	0.9169	0.9940
MCV	1	2	0.006614	0.03373	0.20	0.8445	0.9790
MCV	1	0	0.02887	0.03578	0.81	0.4198	0.6988
MCV	2	0	0.02225	0.03578	0.62	0.5340	0.8081
MCHC	1	2	0.02000	0.01799	1.11	0.2662	0.5067
MCHC	1	0	-0.01331	0.01908	-0.70	0.4855	0.7650
MCHC	2	0	-0.03331	0.01908	-1.75	0.0808	0.1883
MCH	1	2	0.02765	0.03999	0.69	0.4893	0.7685
MCH	1	0	0.01527	0.04242	0.36	0.7189	0.9311
MCH	2	0	-0.01238	0.04242	-0.29	0.7703	0.9541
TPP	1	2	-0.2016	0.2363	-0.85	0.3037	0.6700
TPP	1	0	-0.3842	0.2507	-1.53	0.1253	0.2755
TPP	2	0	-0.1827	0.2507	-0.73	0.4662	0.7465
RDW	1	2	-0.03441	0.02608	-1.32	0.1870	0.3843
RDW	1	0	-0.01010	0.02767	-0.37	0.7150	0.9291
RDW	2	0	0.02431	0.02767	0.88	0.3795	0.6537
Plaquets	1	2	-0.1220	0.2091	-0.58	0.5594	0.8288
Plaquets	1	0	-0.3171	0.2218	-1.43	0.1527	0.3254
Plaquets	2	0	-0.1951	0.2218	-0.88	0.3790	0.6531

Group 0 (G0) - control group, before brain death. Group 1 - group immediately after brain death induction. Group 2 - group 1 hour after brain death induction. The level of significance adopted for the statistical tests was 5% (p<0.05).

**Figure 3.**
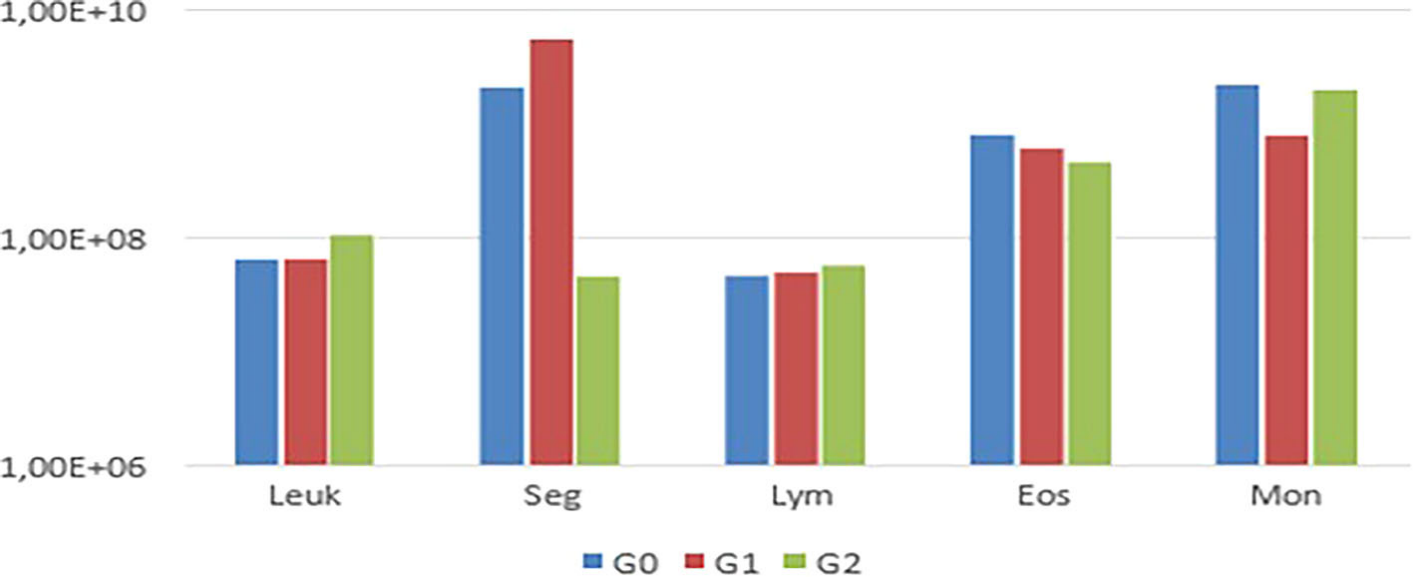
Comparison of mean leukogram values among G0, G1, and G2, showing variations in segmented leukocytes and monocytes.

Notably, animals in G1 exhibited thrombocytopenia. In the leukogram ( Table 2), comparisons among G0, G1, and G2 regarding total leukocytes, segmented leukocytes, lymphocytes, eosinophils, and monocytes from the experimental animals revealed variations in segmented leukocytes and monocytes (p<0.05) ( Figure 3).

**Table 2.** Comparisons between groups 0, 1 and 2 of the values of Total Leukocytes, Segmented Leukocytes, Lymphocytes, Eosinophils and Monocytes of the Leukogram obtained from the experimental animals. Differences in group least squares means, adjusted for multiple comparisons - Tukey-Kramer of the variables.

Variable	Group	_Group	Estimate	Standart Error	Value z	Pr˃│z│	Adj p
Leukocytes	1	2	-0.4788	0.3086	-1.55	0.1208	0.2670
Leukocytes	1	0	0.02027	0.3273	0.06	0.9506	0.9979
Leukocytes	2	0	0.4991	0.3273	1.52	0.1273	0.2793
Segmented	1	2	4.8179	0.8869	5.43	<.0001	<.0001 *
Segmented	1	0	0.9925	0.9407	1.06	0.2913	0.5420
Segmented	2	0	-3.8245	0.9407	-4.07	<.0001	0.0001 *
Lymphocytes	1	2	-0.1416	0.3453	-0.41	0.6816	0.9114
Lymphocytes	1	0	0.06694	0.3662	0.18	0.8550	0.9817
Lymphocytes	2	0	0.2086	0.3662	0.57	0.5690	0.8363
Eosinophils	1	2	0.2800	0.3098	0.90	0.3660	0.6378
Eosinophils	1	0	-0.4601	0.3463	-1.33	0.1841	0.3794
Eosinophils	2	0	-0.7401	0.3463	-2.14	0.0326	0.0825
Monocytes	1	2	-0.9122	0.3619	-2.52	0.0117	0.0314 *
Monocytes	1	0	-1.0284	0.3838	-2.68	0.0074	0.0202 *
Monocytes	2	0	-0.1162	0.3838	-0.30	0.7621	0.9607

Group 0 (G0) - control group, before brain death. Group 1 - group immediately after brain death induction. Group 2 - group 1 hour after brain death induction. The level of significance adopted for the statistical tests was 5% (p<0.05).

* Substantial statistical difference (when p<0.05).

**Figure 4.**
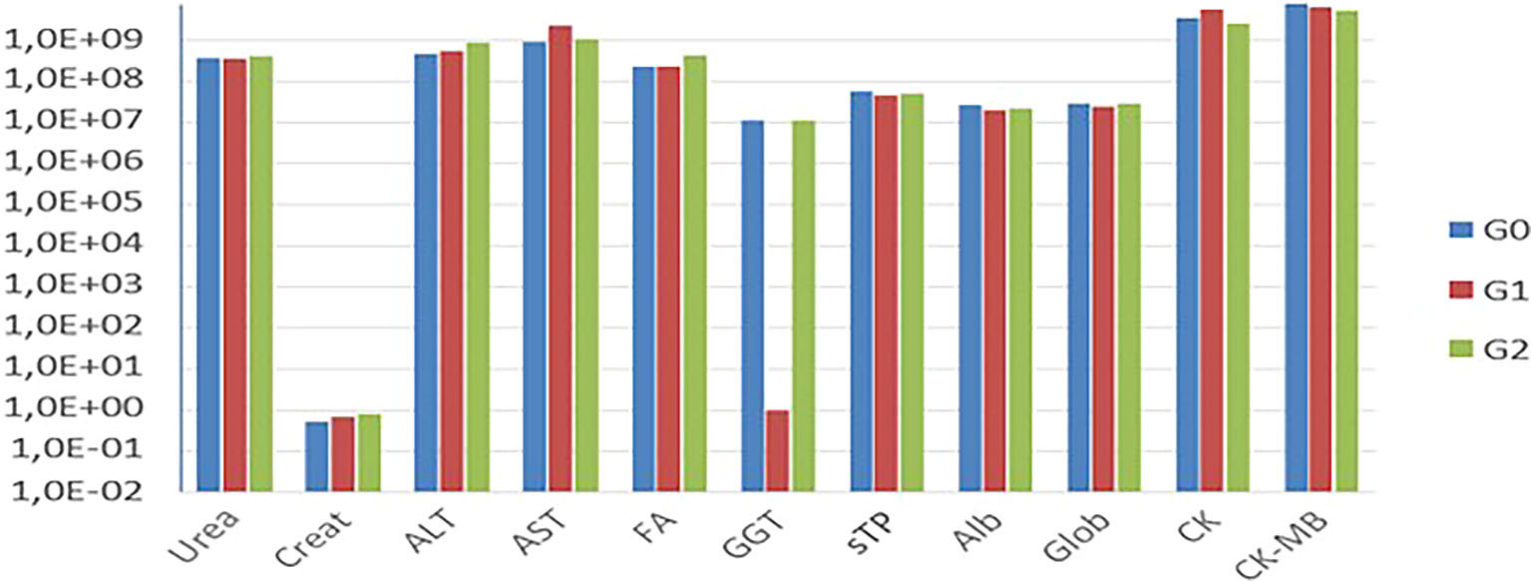
Comparison of mean values of biochemical tests among G0, G1, and G2.

The biochemical tests ( Table 3) compared G0, G1, and G2 regarding urea, Cr, ALT, AST, ALP, GGT, serum TP, albumin, globulin, CK, and CK-MB from the experimental animals ( Figure 4). Only Cr and AST exhibited variations in their values.

**Table 3.** Comparison between groups 0, 1 and 2 in relation to the values of Urea, Creatinine, Alanine Aminotransferase (ALT), Aspartate Aminotransferase (AST), Alkaline Phosphatase (AF), Gamma Glutamyl Transferase (GGT), Total Serum Protein (TSP), Albumin, Globulin, Creatine Kinase (CK) and Creatine Kinase - MB (CK-MB) of the Biochemical Examination obtained from the experimental animals. Differences in group least squares means, adjusted for multiple comparisons - Tukey-Kramer of the variables.

Variable	Group	_Group	Estimate	Standart Error	Value z	Pr˃│z│	Adj p
Urea	1	2	-0.05071	0.1513	-0.34	0.7375	0.9400
Urea	1	0	0.07189	0.1513	0.48	0.6348	0.8831
Urea	2	0	0.1226	0.1513	0.81	0.4179	0.6967
Creatinine	1	2	-0.1210	0.1460	-0.83	0.4071	0.6850
Creatinine	1	0	0.2935	0.1460	2.01	0.0444	0.1096
Creatinine	2	0	0.4146	0.1460	2.84	0.0045	0.0126 *
ALT	1	2	-0.3916	0.2849	-1.37	0.1692	0.3542
ALT	1	0	0.2354	0.2849	0.83	0.4087	0.6867
ALT	2	0	0.6270	0.2849	2.20	0.0277	0.0710
AST	1	2	0.7952	0.3469	2.29	0.0219	0.0568
AST	1	0	0.9538	0.3469	2.75	0.0060	0.0164 *
AST	2	0	0.1586	0.3469	0.46	0.6474	0.8911
AF	1	2	-0.5406	0.2725	-1.98	0.0472	0.1161
AF	1	0	0.08733	0.2725	0.32	0.7486	0.9449
AF	2	0	0.6280	0.2725	2.30	0.0212	0.0551
GGT	1	2	-0.06188	0.05020	-1.23	0.2177	0.4339
GGT	1	0	-0.06188	0.05020	-1.23	0.2177	0.4339
GGT	2	0	1.03E-17	0.05020	0.00	1.0000	1.0000
TSP	1	2	-0.06744	0.1127	-0.60	0.5497	0.8210
TSP	1	0	-0.1588	0.1127	-1.41	0.1589	0.3364
TSP	2	0	-0.09135	0.1127	-0.81	0.4177	0.6966
Albumin	1	2	-0.01026	0.1774	-0.06	0.9539	0.9982
Albumin	1	0	-0.2044	0.1774	-1.15	0.2493	0.4821
Albumin	2	0	-0.1942	0.1774	-1.09	0.2739	0.5177
Globulin	1	2	-0.1121	0.08407	-1.33	0.1824	0.3765
Globulin	1	0	-0.1197	0.08407	-1.42	0.1546	0.3288
Globulin	2	0	-0.00755	0.08407	-0.09	0.9285	0.9956
CK	1	2	0.8398	0.5325	1.58	0.1148	0.2556
CK	1	0	0.5379	0.5325	1.01	0.3125	0.5705
CK	2	0	-0.3019	0.5325	-0.57	0.5707	0.8377
CK-MB	1	2	0.2818	0.5311	0.53	0.5956	0.8563
CK-MB	1	0	-0.1141	0.5311	-0.21	0.8299	0.9749
CK-MB	2	0	-0.3959	0.5311	-0.75	0.4559	0.7363

Group 0 (G0) - control group, before brain death. Group 1 - group immediately after brain death induction. Group 2 - group 1 hour after brain death induction. The level of significance adopted for the statistical tests was 5% (p<0.05).

* Substantial statistical difference (when p<0.05).

Overall, blood gas analysis ( Table 4) showed that most animals in all groups had low hydrogen potential (pH) levels, increased oxygen partial pressures (PaO _2_), and carbon dioxide partial pressures (PaCO _2_).

**Table 4.** Analysis of values of the Gasometric test variables of the animals in each group.

Gasometric											
A	G	pH	pCO _2_	pO _2_	Na ^+^	K ^+^	Ca ^2+^	Cl ^-^	Lac	HCO _3_	sO _2_
1	0	6,97	132,7	142	152	4,16	0,93	108	1,2	29,1	96,7
2	0	7,09	82,0	99	153	4,63	0,43	110	2,9	24,0	93,9
3	0	7,24	63,2	170	147	4,74	0,72	117	1,3	26,4	99,2
4	0	7,10	78,9	180	149	4,81	0,85	109	5,2	23,1	98,9
5	0	7,16	86,1	168	137	4,89	0,90	108	0,6	29,7	98,9
6	1	6,92	91,0	157	154	5,16	1,07	103	5,3	17,8	97,3
7	1	7,24	41,0	63	145	5,43	0,93	110	7,4	17,0	87,7
8	1	7,12	69,7	208	149	4,65	0,79	111	4,7	17,7	99,4
9	1	6,97	70,9	187	151	4,82	0,95	104	6,9	15,6	98,7
10	1	7,23	40,8	45	127	6,22	0,49	101	4,6	16,4	73,0
11	2	6,74	75,9	143	154	8,23	0,72	94	9,5	9,8	94,3
12	2	7,08	70,4	157	156	4,61	0,58	112	0,5	20,1	98,4
13	2	7,03	56,6	134	151	6,11	1,13	104	6,2	14,4	97,2
14	2	7,16	87,4	124	144	5,69	1,03	108	1,2	30,0	97,3
15	2	7,12	77,7	64	150	4,75	1,14	110	0,8	24,3	82,8

A: Animal, G: Group, pH: blood hydrogen potential, pCO2: partial pressure of carbon dioxide (mmHg), pO _2_: partial pressure of oxygen (mmHg), Na+: Sodium (mmol/L), K ^+^: Potassium (mmol/L), Ca ^+^: Calcium (mmol/L), Cl ^-^: Chlorine (mmol/L), Lac: Lactate (mmol/L), HCO _3_: Bicarbonate (P), sO _2_: oxygen saturation index (%). N: number of samples, Group 1 - group immediately after brain death induction. Group 2 - group 1 h after induction of brain death.

In some animals, bicarbonate (HCO _3_) levels were decreased, and the oxygen saturation index (sO _2_) and lactate values tended to increase. Moreover, sodium levels showed a tendency to increase in most animals, potassium increased in only one, calcium decreased uniformly across all, and chloride tended to rise in the majority.

In the bivariate analysis, the initial analysis of the nine erythrogram variables in group 0 using the Spearman test showed positive correlations with hemoglobin (Hb), platelets, mean corpuscular volum (MCV), and mean corpuscular hemoglobin (MCH) ( Tables 5 and 6).

**Table 5.** Descriptive statistics of the Erythrogram variables of Group 0.

Simple statistics						
Variable	N	Mean	Standard Deviation	Median	Minimum	Maximum
**RBC**	4	8.69250	0.86904	8.69000	7.86000	9.53000
**Hb**	4	16.85000	0.69522	16.85000	16.00000	17.70000
**HCT**	4	48.50000	3.10913	47.50000	46.00000	53.00000
**MCV**	4	56.00000	3.57678	56.45000	51.30000	59.80000
**MCHC**	4	34.80000	1.07083	34.90000	33.40000	36.00000
**MCH**	4	19.50000	1.55134	19.25000	18.00000	21.50000
**TPP**	4	6.05000	0.30000	6.20000	5.60000	6.20000
**RDW**	4	14.42500	0.76757	14.40000	13.60000	15.30000
**Platelets**	4	865.44375	136.30908	879.96250	707.00000	994.85000

RBC: Red Blood Cells ( $10^{6}$ /μL), Hb: Hemoglobin (g/dL), HCT: Hematocrit (%), MCV: Mean Corpuscular Volume (fL), MCHC: Mean Corpuscular Hemoglobin Concentration (%), MCH: Mean Corpuscular Hemoglobin (pg), TPP: Total Plasma Protein (%), RDW: Red Cell Distribution Width (%), Platelets (/μL).

**Table 6.** Spearman's Correlation between the Erythrogram variables in Group 0.

Spearman's Correlation Coefficients (N = 4) Prob >|r|sob H0: Rho=0									
	RBC	Hb	HCT	MCV	MCHC	MCH	TPP	RDW	Platelets
** RBC**	1.00000	0.40000	0.80000	-0.80000	-0.80000	-0.80000	0.25820	0.20000	0.40000
		0.6000	0.2000	0.2000	0.2000	0.2000	0.7418	0.8000	0.6000
**Hb**	0.40000	1.00000	0.80000	0.00000	-0.20000	0.00000	0.77460	-0.80000	1.00000
	0.6000		0.2000	1.0000	0.8000	1.0000	0.2254	0.2000	<.0001 *
**HCT**	0.80000	0.80000	1.00000	-0.60000	-0.40000	-0.60000	0.77460	-0.40000	0.80000
	0.2000	0.2000		0.4000	0.6000	0.4000	0.2254	0.6000	0.2000
**MCV**	-0.80000	0.00000	-0.60000	1.00000	0.40000	1.00000	-0.25820	-0.40000	0.00000
	0.2000	1.0000	0.4000		0.6000	<.0001 *	0.7418	0.6000	1.0000
**MCHC**	-0.80000	-0.20000	-0.40000	0.40000	1.00000	0.40000	0.25820	-0.40000	-0.20000
	0.2000	0.8000	0.6000	0.6000		0.6000	0.7418	0.6000	0.8000
**MCH**	-0.80000	0.00000	-0.60000	1.00000	0.40000	1.00000	-0.25820	-0.40000	0.00000
	0.2000	1.0000	0.4000	<.0001 *	0.6000		0.7418	0.6000	1.0000
**TPP**	0.25820	0.77460	0.77460	-0.25820	0.25820	-0.25820	1.00000	-0.77460	0.77460
	0.7418	0.2254	0.2254	0.7418	0.7418	0.7418		0.2254	0.2254
**RDW**	0.20000	-0.80000	-0.40000	-0.40000	-0.40000	-0.40000	-0.77460	1.00000	-0.80000
	0.8000	0.2000	0.6000	0.6000	0.6000	0.6000	0.2254		0.2000
**Platelets**	0.40000	1.00000	0.80000	0.00000	-0.20000	0.00000	0.77460	-0.80000	1.00000
	0.6000	<.0001 *	0.2000	1.0000	0.8000	1.0000	0.2254	0.2000	

RBC: Red Blood Cells ( $10^{6}$ /μL), Hb: Hemoglobin (g/dL), HCT: Hematocrit (%), MCV: Mean Corpuscular Volume (fL), MCHC: Mean Corpuscular Hemoglobin Concentration (%), MCH: Mean Corpuscular Hemoglobin (pg), TPP: Total Protein (%), RDW: Red Cell Distribution Width (%), Platelets (/μL).

* Substantial statistical difference (when p<0.05).

## Discussion

The meticulous control and interpretation of laboratory test changes are intrinsic to diagnosing BD. ^11, 12^ Individual examination within each group revealed a tendency towards polycythemia in most animals, particularly notable in G2, consistent with findings from Fiocruz. ^13^ This can be explained by hypoxemia resulting from BD, leading to secondary absolute polycythemia. One subject in G1 showed anemia and hypoproteinemia. One study found hemoglobin and hematocrit concentrations also decreased after BD induction. ^7^ There were also animals in this group that showed thrombocytopenia. Another research revealed 38.5% of organ donors exhibited anemia and 30.8% had thrombocytopenia. ^28^ This phenomenon is attributed to common coagulation disorders following BD, driven by factors such as thromboplastin release, damaged brain tissue fibrinogen release, disseminated intravascular coagulation (DIC), and platelet and coagulation factor consumption due to fluid resuscitation volumes. ^12^

Regarding the analysis of variables in the leukogram examination, the reduction in segmented neutrophils in G2 may be related to increased bone marrow immunosuppression 1 h after BD. An increase in the number of circulating hematopoietic precursor cells, followed by bone marrow dysfunction, has been observed in patients with severe trauma, hemorrhagic shock, or burns. Stroke induces systemic inflammatory response and subsequent immunosuppression. ^14, 15^

Evaluating the animals in each group individually, two animals (A6 and A11) in G1 and G2, respectively, presented leukocytosis. This change is justified by the inflammatory state resulting from the BD process, which activates inflammatory mediators like thromboxanes and leukocyte factors. Among the potential human donors, 66.2% had leukocytosis. ^16, 17^ Conversely, Menegat and Sannomiya observed BD-induced leukopenia in rats, resulting in a reduction in lymphocytes, monocytes, and granulocytes. ^18^

The difference in Cr values between G0 and G2 in this study may be associated with renal function failure due to hemodynamic instability and hypotension, leading to decreased perfusion. ^19^ Another factor that can affect the kidneys and cause a sudden elevation of plasma Cr, which is widely used as an indicator of acute renal failure and changes in diuresis volume, is rhabdomyolysis. ^20^ In this study, most of the gas tests presented an acid-base imbalance. Most animals tended towards respiratory acidosis, and some presented metabolic acidosis. These disorders are associated with an increased risk of organ and system dysfunction, and metabolic acidosis is an indicator of unfavorable clinical outcomes. ^21, 22^

In BD, gasometry values vary considerably, as the lungs are among the first organs to suffer changes in the body. The lungs seek to compensate and preserve a physiologically acceptable pH when the renal ability to maintain homeostasis is lost. ^23^ However, the lung tissue can be affected by pathophysiological and endocrine changes and the inflammatory reactions caused by BD. ^24^ Additionally, perfusion-ventilation imbalance and hypoxemia are the main manifestations of pulmonary changes due to intense adrenergic discharge, leading to increased venous return to the right ventricle consequently increased pulmonary flow. Along with elevated left atrial pressure from intense peripheral vasoconstriction, this results in increased capillary hydrostatic pressure, promoting capillary rupture, interstitial edema, and alveolar hemorrhage. ^25^

Abnormal partial oxygen pressure promotes the release of tumor necrosis factor-alpha and IL-1 beta, which are inflammatory cytokines that mediate lung lesions. ^20^ According to Mascia et al.19 and Botha et al.,6 30–45% of potential donors develop lung injury, with acute lung injury (ALI) or acute respiratory distress syndrome (ARDS) being the most frequent. Nogueira and Pereira25 observed that 69.2% of patients had hyperoxia due to oxidative stress in the clinical picture of BD. In this study, lactate levels tended to increase. This is mainly due to altered energy metabolism, where excessive prolongation of anaerobic metabolism overcomes the body’s ability to remove lactate, leading to metabolic acidosis, which can become severe and cause fatigue, even when buffering mechanisms are well-developed. ^26, 27^ According to Hodgson and Rose, an anaerobic threshold is commonly reached when lactate. ^28^ concentration is between 2–4 mmol/L. In our study, higher lactate levels were observed.

In most animals, hypernatremia was observed, and an increase in potassium was seen in only one animal, contradicting Westphal et al., ^19^ who observed hyperkalemia and hypomagnesemia as common disorders associated with BD, potentially leading to arrhythmia. Additionally, calcium decreased in all animals, whereas chlorine levels tended to increase in most cases. Hypernatremia was observed in most animals, and an increase in potassium was seen in only one animal. This contradicts Westphal et al., ^19^ be related to progressive dysfunction of the hypothalamic-pituitary axis after BD and, in 80% of cases, the development of diabetes insipidus, which results in a decline in circulating antidiuretic hormone. ^29^ The polyuria resulting from this endocrine/metabolic change can lead to serious electrolyte disorders, such as hypernatremia, hypokalemia, hypocalcemia, hypophosphatemia, and hypomagnesemia. ^13^ In a study by Vasconcelos et al. with possible human donors, 47.7% had hypernatremia, similar to the values obtained in this study. ^30^

Therefore, this study recognizes and highlights the deleterious effects caused by BD, noting that these effects are multiple and cause various complications in the body, so an evaluation using different combined analyses is important, as shown in this study, to also diagnose different critical diseases prior to the brain death itself. Early detection is important to avoid progressive somatic deterioration and ensure better organ function. ^30, 31^ The experimental model used in this study, which is interesting for evaluating a short period of time, did not produce more significant results for many variables. The speed of diagnosing BD may not lead to major changes in tests within 1 h after BD. Additionally, the number of animals (n) used was a limiting factor. In conclusion, BD showed a unique response in each organism, complicating its precise determination even more.

## Ethics

We confirm that all efforts were made to ameliorate suffering of animals applying the rules settled to animal pain management and suffering in the “Brazilian Guide to Good Animal Euthanasia Practices” (available online in: https://www.cfmv.gov.br/guia-brasileiro-de-boas-praticas-para-a-eutanasia-em-animais/comunicacao/publicacoes/2020/08/03/#1) dictated by the Brazilian Federal Council of Veterinary Medicine (CFMV). Ethical Principles in Animal Research adopted by the Brazilian College of Animal Experimentation (COBEA) and were approved by the Ethics Committee on the Use of Animals (CEUA) of the Faculty of Medicine (FMB) of UNESP, Botucatu Campus, protocol No. 0259/2018 on 16 ^th^ January 2019.

## Data Availability

Figshare: Parâmetros hematológicos, gasométricos e bioquímicos na indução da morte encefálica em ratos Wistar, DOI: https://doi.org/10.6084/m9.figshare.27586602.v6 ^32^ This project contains the following underlying data:
• PARÂMETROS HEMATOLÓGICOS, GASOMÉTRICOS E.docx • Underlying data spreadsheet.xlsx • IACUC •
mazzante_nmg_dr_bot_sub-english.docx • Underlyingdataspreadsheet-english.xlsx • ARRIVE Checklist Data are available under the terms of the Creative Commons Attribution 4.0 International license (CC-BY 4.0). **Figshare:** Arrive checklist for ***‘*** *Parâmetros hematológicos, gasométricos e bioquímicos na indução da morte encefálica em ratos Wistar’*, DOI: https://doi.org/10.6084/m9.figshare.27586602.v6 Data are available under the terms of the Creative Commons Attribution 4.0 International license (CC-BY 4.0).
