# Supplementary material for: Diagnosis of brain death in wistar rats at different levels of death induction
Source: F1000Res. 2025 May 6;13:1490. Originally published 2024 Dec 6. [Version 2] doi: 10.12688/f1000research.157233.2 (PMC12082075; doi:10.12688/f1000research.157233.2)
Supplement: Peer review history

# Reviewer report: Reviewer response for version 2

**DOI:** 10.5256/f1000research.181285.r383475

**Published:** 2025-05-24

**Authors:** Calixto Machado

**Affiliations:** 1 Institute of Neurology and Neurosurgery, Havana, Cuba

**General Assessment**

 This manuscript presents an exploratory study on the physiological and biochemical changes associated with brain death (BD) in Wistar rats. It aims to deepen our understanding of the variable responses to BD induction and to identify laboratory markers that may help refine early diagnostic criteria. The subject is highly relevant, particularly given the limited availability of experimental data addressing the pathophysiological dynamics of brain death in preclinical models.

 Despite a modest sample size, the study’s design is clear and methodologically sound. It includes three well-defined groups with varying durations after BD induction and offers statistically supported comparisons of hematologic, biochemical, and gasometric parameters. The observed variations, especially in segmented leukocytes, monocytes, creatinine, AST, potassium, and bicarbonate, contribute to our growing understanding of the systemic impact of BD.

**Ethical and Animal Welfare Considerations**

 Of particular importance is the authors’ adherence to established ethical protocols in animal research. The study was approved by the Ethics Committee on the Use of Animals (CEUA – protocol 0259/2018) and followed both the *Brazilian College of Animal Experimentation (COBEA)* and the *Brazilian Guide to Good Euthanasia Practices*. This adherence ensures that all efforts were made to minimize suffering and prioritize humane treatment throughout the experimental process. The inclusion of environmental enrichment and diligent monitoring further exemplifies best practices in animal care.

 By explicitly detailing the anesthesia protocols, surgical preparation, and post-procedural monitoring, the authors demonstrate a commendable commitment to animal welfare and the 3Rs (Replacement, Reduction, and Refinement) in experimental design.

**Scientific Significance and Recommendations**

 The research contributes novel data on how acute physiological changes manifest post-BD and reinforces the importance of laboratory parameters as early indicators of systemic decline. While the sample size limits generalizability, the study effectively functions as a pilot investigation that could inspire more expansive, statistically powered future research.

 I recommend the **acceptance of this manuscript** with minor revisions aimed at improving the clarity of conclusions and further contextualizing results within existing literature. Expanding on the potential clinical translational value of such findings in veterinary and human medicine would be particularly beneficial.

**Final Recommendation:**

 ✅ *Accept with minor revisions*

*This study is ethically sound, scientifically relevant, and presents well-supported preliminary findings that merit dissemination.*

Is the work clearly and accurately presented and does it cite the current literature?

Yes

If applicable, is the statistical analysis and its interpretation appropriate?

Yes

Are all the source data underlying the results available to ensure full reproducibility?

Yes

Is the study design appropriate and is the work technically sound?

Yes

Are the conclusions drawn adequately supported by the results?

Yes

Are sufficient details of methods and analysis provided to allow replication by others?

Yes

Reviewer Expertise:

Brain death, disorders of consciousness, neuroimaging, general neurology

I confirm that I have read this submission and believe that I have an appropriate level of expertise to confirm that it is of an acceptable scientific standard.

# Reviewer report: Reviewer response for version 2

**DOI:** 10.5256/f1000research.181285.r383095

**Published:** 2025-05-14

**Authors:** Chiara Lazzeri

**Affiliations:** 1 Careggi Hospital, Florence, Italy

I read the revised version of the manuscript. The Authors provided a point to point reply to the reviewers' comments. The manuscript improved.

Is the work clearly and accurately presented and does it cite the current literature?

Partly

If applicable, is the statistical analysis and its interpretation appropriate?

Yes

Are all the source data underlying the results available to ensure full reproducibility?

Partly

Is the study design appropriate and is the work technically sound?

Yes

Are the conclusions drawn adequately supported by the results?

Partly

Are sufficient details of methods and analysis provided to allow replication by others?

Yes

Reviewer Expertise:

organ procurement and transplantation, cardiac arrest, ECMO, respiratory failure, critical care echocardiography

I confirm that I have read this submission and believe that I have an appropriate level of expertise to confirm that it is of an acceptable scientific standard.

# Reviewer report: Reviewer response for version 2

**DOI:** 10.5256/f1000research.181285.r383096

**Published:** 2025-05-13

**Authors:** Mario Abbud-Filho

**Affiliations:** 1 Medicine, Hospital de Base (FUNFARME) and Medical School (FAMERP), São José do Rio Preto, São Paulo, Brazil

The authors answered the questions we addressed and improved the revised version. In the interest of the manuscript's clarity, they should include a visual representation, such as a diagram or timeline, summarizing the experimental design, particularly the timing of sample collection across the different groups. The illustration could make the description clearer and more concise. 

 We thank you for the opportunity to revise the manuscript.

Is the work clearly and accurately presented and does it cite the current literature?

Partly

If applicable, is the statistical analysis and its interpretation appropriate?

I cannot comment. A qualified statistician is required.

Are all the source data underlying the results available to ensure full reproducibility?

Yes

Is the study design appropriate and is the work technically sound?

Partly

Are the conclusions drawn adequately supported by the results?

Partly

Are sufficient details of methods and analysis provided to allow replication by others?

Partly

Reviewer Expertise:

Renal transplantation, donor preconditioning, molecular aspects of marginal kidneys

I confirm that I have read this submission and believe that I have an appropriate level of expertise to confirm that it is of an acceptable scientific standard.

# Reviewer report: Reviewer response for version 1

**DOI:** 10.5256/f1000research.172655.r366145

**Published:** 2025-03-04

**Authors:** Mario Abbud-Filho, Ludmila Marzochi

**Affiliations:** 1 Medicine, Hospital de Base (FUNFARME) and Medical School (FAMERP), São José do Rio Preto, São Paulo, Brazil

I was asked to review the manuscript entitled *“Diagnosis of Brain Death in Wistar Rats at Different Levels of Death Induction”* authored by Mazzante et al.

 Although there is indeed a debate—more philosophical than medical—about the definition of brain death, in the field of transplantation, there is no doubt regarding the selection of potential organ donors. From a biochemical and molecular perspective, brain death is of particular interest to researchers in this field, making the authors' work relevant.

 However, several issues raise major concerns:

1. The small sample size limits the generalizability of the findings. Although the authors acknowledge this limitation, it remains a significant constraint.
2. The selected time points for collecting laboratory parameters (M1 and M2) are difficult to interpret, as they primarily reflect acute changes associated with brain death.
3. It seems overly simplistic to conclude the highly complex process of brain death based solely on the chosen laboratory parameters (hematological, renal, and hepatic markers).
4. While the study identifies significant changes in various parameters, it does not sufficiently explore the underlying mechanisms driving these changes. For instance, the discussion could further elaborate on how brain death-induced inflammatory responses or hormonal imbalances contribute to the observed alterations in hematologic and biochemical markers.
5. The discussion section could be strengthened by addressing conflicting findings in the literature more thoroughly. For example, while the study reports hypernatremia and hypokalemia, potential reasons for these discrepancies could have been explored more deeply.
6. The conclusion is vague and does not fully summarize the key findings or their implications. A more concise and impactful conclusion would enhance the manuscript.

**In summary,** the study is scientifically sound and addresses a relevant topic. However, significant revisions are necessary to improve its clarity, depth, and overall impact.

Is the work clearly and accurately presented and does it cite the current literature?

Partly

If applicable, is the statistical analysis and its interpretation appropriate?

I cannot comment. A qualified statistician is required.

Are all the source data underlying the results available to ensure full reproducibility?

Yes

Is the study design appropriate and is the work technically sound?

Partly

Are the conclusions drawn adequately supported by the results?

Partly

Are sufficient details of methods and analysis provided to allow replication by others?

Partly

Reviewer Expertise:

Renal transplantation, donor preconditioning, molecular aspects of marginal kidneys

We confirm that we have read this submission and believe that we have an appropriate level of expertise to state that we do not consider it to be of an acceptable scientific standard, for reasons outlined above.

# Response

**Published:** 2025-04-27

**Authors:** Fernanda Barthelson Carvalho de Moura

It is with great enthusiasm that we respond to  Dr. Mario Abbud-Filho Dr. Ludmila Marzochi and Dr. Chiara Lazzeri comments concerning the study “Diagnosis of brain death in wistar rats at different levels of death induction” ^1^. The authors genuinely appreciate this consideration and interest in our work.

 In response to reviewers’ comments and requests, the time criteria used in the research  follow the main goal of this research as we aim to evaluate the brain death in different levels of death induction.  Using these criteria, we were able to evaluate de progression of the parameters in different times and understand how the brain death progress, from immediately after death, hour after death compared with our control group. According to Hannodee and Nasuruddin, the acute changes manifest immediately after injury and can last for a few days ^2^.

 Additionally, the laboratory parameters play a fundamental role in understanding the progression of brain death, as death results in leakage of cytokines, proteins, and other components, mainly in the acute phase.  Also, several changes in the brain death may not be detectable in macroscopical examination, which is the reason why the study primarily focused on laboratory parameters, which could provide early indications of physiological changes.

 We want to enhance that the primary objective of this paper was to evaluate the hematologic, biochemical, and gasometrical parameters in brain death to understand the modifications and reduce misdiagnosis by using these parameters as an indication in early stages.  We acknowledge that discussing those changes could be explored in more detail. However, the experimental nature of this paper should be considered, as well as the small number of animals used in this research. Our results, even in a short interval time evaluated and with a short number of animal models, demonstrate the tendency of those changes. Further studies with more animals and extended observation periods are necessary to elaborate a more comprehensive discussion of the mechanisms involved.

 The authors agree that the discussion could be more detailed and deeper.  However, our research is experimental, and we performed an individual examination of the specific groups used in the research. We provided a discussion comparing our findings with other results in the literature (ie, Fiocruz, Hodgson, and Rose) ^3,4^; however, this type of study, in which brain death is evaluated by animal laboratory changes, is not well studied and explored. We hope our study stimulates further research, which could provide a better discussion of the results.

 In conclusion we believe that this study highlights the complex physiological changes associated with brain death (BD) in Wistar rats. Significant differences were observed between groups in the laboratory analysis, such as: segmented cells, monocytes, creatinine, aspartate aminotransferase, potassium, and bicarbonate levels, demonstrating the varied impact of BD on these parameters. The study shows that early detection is important to avoid progressive somatic deterioration and ensure better organ function. Although the experimental model and sample size in this work were not ideal, it has the potential to contribute to the understanding that individual responses to BD oscillate, complicating its diagnosis and emphasizing the need for further investigation to improve clinical diagnostic precision.

 We sincerely wish we could now have explored these points more in our response and paper. In closing, we sincerely thank Dr. Mario Abbud-Filho, Dr. Ludmila Marzochi, and Dr. Chiara Lazzeri for providing an opportunity to clarify our perspective and initiating a professional, productive, and interesting discussion.

 References

1. Gil Mazzante NM, Zuliani F, Oliveira RAd et al. Diagnosis of brain death in wistar rats at different levels of death induction [version 1; peer review: 1 approved with reservations, 1 not approved]. F1000Research 2024, 13:1490 ( https://doi.org/10.12688/f1000research.157233.1
2. Hannoodee S, Nasuruddin DN. Acute Inflammatory Response. 2024 Jun 8. In: StatPearls [Internet]. Treasure Island (FL): StatPearls Publishing; 2025 Jan–. PMID: 32310543.
3. Nogueira EC: Captação de órgãos em Sergipe e fatores associados à efetivação de potenciais doadores; 2008. 103f. Dissertação (Mestrado) - Universidade Federal de Sergipe. Aracaju.
4. Hodgson DR, Rose RJ: *The athletic horse: principles and practice of equine sports medicine.* 1st ed.Philadelphia: W. B. Saunders Company; 1994; 245–258.

# Reviewer report: Reviewer response for version 1

**DOI:** 10.5256/f1000research.172655.r347400

**Published:** 2024-12-17

**Authors:** Chiara Lazzeri

**Affiliations:** 1 Careggi Hospital, Florence, Italy

The topic is quite interesting. The main limitation of the present study is the small number of animals included. This should be addressed in the discussion section. 

 Major issues can be raised:

 a) in the discussion section each altered parameter is described and discussed. Regarding polycytemia, other hypothesis should be considered, especially volemia

 b) in regard to coagulation disorders, Authors should add other hypothesized mechanism(s), including the cause of brain death (i.e. trauma)

 c) what about treatment of brain death

 d) the Authors should better elucidate the novelty of their investigation in respect to existing evidence on this topic

Is the work clearly and accurately presented and does it cite the current literature?

Partly

If applicable, is the statistical analysis and its interpretation appropriate?

Yes

Are all the source data underlying the results available to ensure full reproducibility?

Partly

Is the study design appropriate and is the work technically sound?

Yes

Are the conclusions drawn adequately supported by the results?

Partly

Are sufficient details of methods and analysis provided to allow replication by others?

Yes

Reviewer Expertise:

organ procurement and transplantation, cardiac arrest, ECMO, respiratory failure, critical care echocardiography

I confirm that I have read this submission and believe that I have an appropriate level of expertise to confirm that it is of an acceptable scientific standard, however I have significant reservations, as outlined above.
